# Crystal structure and metabolic activity of 4-(thien-2-yl)-2-methyl-5-oxo-1,4,5,6,7,8-hexa­hydro­quinoline-3-carb­oxy­lic acid eth­oxy­carbonyl­phenyl­methyl­ester

**DOI:** 10.1107/S2056989018014251

**Published:** 2018-10-12

**Authors:** Anatoly Mishnev, Egils Bisenieks, Ilona Mandrika, Ramona Petrovska, Zenta Kalme, Imanta Bruvere, Gunars Duburs

**Affiliations:** a Latvian Institute of Organic Synthesis, Aizkraukles Str. 21, Riga, LV-1006, Latvia; b Latvian Biomedical Research and Study Centre, Ratsupites Str. 1, Riga, LV-1067, Latvia

**Keywords:** crystal structure, 1,4-di­hydro­pyridine, metabolic activity, disorder

## Abstract

In the title compound, C_25_H_25_NO_5_S, which exhibits metabolism-regulating activity, the 1,4-di­hydro­pyridine ring adopts a flattened boat conformation while the cyclo­hexenone ring is in an envelope conformation. Mol­ecules in the crystal are assembled into *C*(6) chains along the *a*-axis direction *via* N—H⋯O hydrogen bonds. The thienyl fragment is disordered over two sets of sites in a 0.7220 (19):0.2780 (19) ratio.

## Abstract 2

In this condensed 1,4-di­hydro­pyridine derivative, which exhibits metabolism-regulating activity, the 1,4-di­hydro­pyridine ring adopts a flattened boat conformation while the cyclo­hexenone ring is in an envelope conformation. Mol­ecules in the crystal are assembled into chains along the *a*-axis direction *via* N—H⋯O hydrogen bonds.

## Chemical context   

Up to now, the 2-methyl-5-oxo-1,4,5,6,7,8-hexa­hydro­quino­line-3-carb­oxy­lic esters in the class of condensed 1,4-di­hydro­pyridine (DHP) derivatives have been relatively poorly studied. Monocyclic DHPs are very commonly known as cardiovascular regulating and hypotensive compounds (Swarnalatha *et al.*, 2011 ▸). The title compound is an original substance with a specific ligand effect on the metabolism-regulating free fatty acid receptor 3 (FFAR3 or GPR41). At the same time, it does not act on other metabolite-sensing receptors such as FFAR2 (GPR43) or the hy­droxy­carb­oxy­lic receptor 2 (HCA2) having similar pharmacological effects.
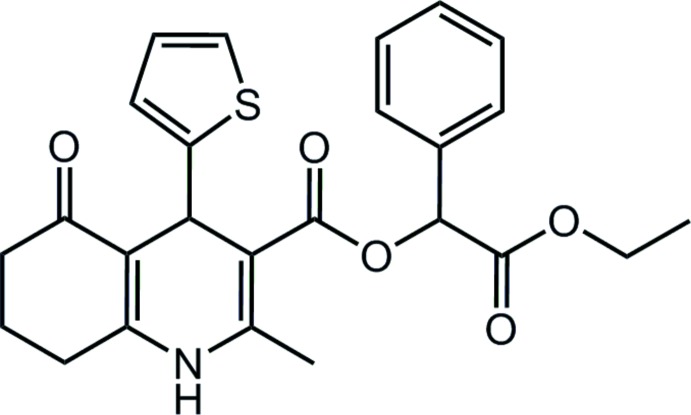


## Structural commentary   

**Figure 1:**
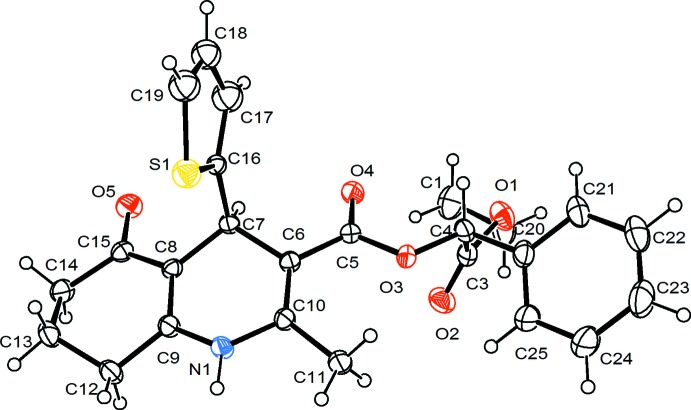
The mol­ecular structure of the title compound with the atom-numbering scheme and 50% probability displacement ellipsoids. Only the major component of the disordered thienyl fragment is shown.

Fig. 1 ▸ shows the mol­ecular structure of the title compound. A two-component disorder is found for the thienyl fragment, which assumes two orientations differing by a 180° rotation around the C7—C16 bond. The major component has a refined occupancy of 0.7220 (19) and is that shown in Fig. 1 ▸. The 1,4-di­hydro­pyridine ring adopts a flattened boat conformation while the cyclo­hexenone ring is in an envelope conformation. Atoms C7 and N1 deviate by 0.298 (3) and 0.135 (3) Å, respectively, in the same direction from the mean C6/C8/C9/C10 plane. The fused cyclo­hexene ring has an envelope conformation, with atom C13 out of the C8/C9/C12/C14/C15 plane by 0.628 (3) Å. The thienyl ring is almost perpendicular to the C6/C8/C9/C10 plane, subtending a dihedral angle of 82.50 (8)°.

## Supra­molecular features   

**Figure 2:**
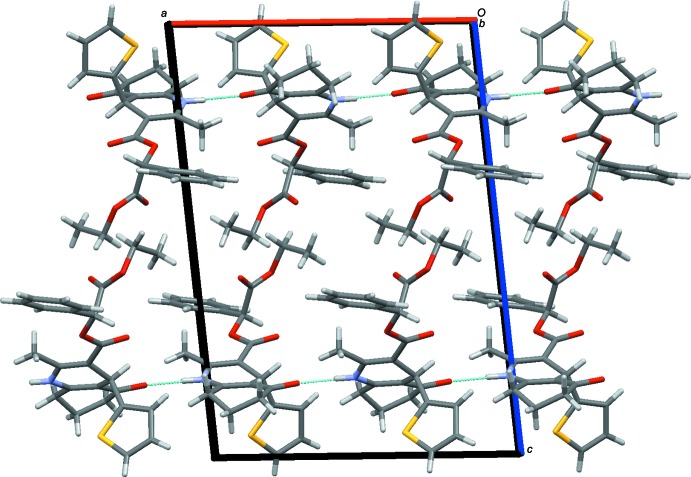
A packing diagram of the title compound, viewed along the *b*-axis direction. N—H⋯O hydrogen bonds are shown as dashed lines.

In the crystal, inter­molecular N—H⋯O hydrogen bonds (Table 1 ▸) assemble the mol­ecules into chains along the *a*-axis direction (Fig. 2 ▸). The hydrogen-bonding pattern in the structure can be described by a *C*(6) graph-set motif. If one denotes the thienyl fragment as the ‘head’ of the mol­ecule and an eth­oxy group as the ‘tail’, then the crystal structure can be described as consisting of head-to-head and tail-to-tail mol­ecular assemblies, or layers, parallel to the *ac* plane and stabilized by van der Waals inter­actions (Fig. 2 ▸).

## Database survey   

A search of the Cambridge Structural Database (CSD Version 5.39, last update February 2018; Groom *et al.*, 2016 ▸) for the 2-methyl-5-oxo-1,4,5,6,7,8-hexa­hydro­quinoline-3-carb­oxy­lic acid fragment gave nine hits: BEZWEK (Kidwai *et al.*, 2012 ▸), FERHEQ (Carroll *et al.*, 2004 ▸), HALLUE (Huang *et al.*, 2016 ▸), JOXTOF (Rose & Dräger, 1992 ▸), LAVWIP (Yu *et al.*, 2005 ▸), RAQROT (Meng *et al.*, 2017 ▸), SUYWIT (Natarajan *et al.*, 2010 ▸), VUZRIS (Yang *et al.*, 2010 ▸) and YIYDUH (Gein *et al.*, 2014 ▸). Unlike the title compound, which has a thienyl group at position 4 of the 1,4-di­hydro­pyridine ring, the most closely related structures found in the CSD have phenyl derivatives exclusively at this position. In all of the selected structures, the 1,4-di­hydro­pyridine ring also assumes a boat conformation of different depths. Deviations from the mean plane of the four basal atoms range from 0.067 (3) to 0.168 (2) Å for the N atom and 0.177 (4) to 0.399 (2) Å for C atoms. The dihedral angles between the planar substituents on the 1,4-di­hydro­pyridine ring and its mean plane are close to 90°. The only exception is LAVWIP with an angle of 83.62 (8)°. Seven of the nine listed crystal structures analogous to the title compound have inter­molecular N—H⋯O-type hydrogen-bonding motifs.

## Metabolic activity   

The title compound possesses considerable and specific activity as a ligand of FFAR3. At 50 µM concentration, the compound inhibits forskolin-stimulated level of cAMP by 60% in recombinant cells expressing FFAR3. The compound through FFAR3 inhibits the cAMP-dependent pathway by inhibiting adenylate cyclase activity and decreasing the production of cAMP, which results in decreased activity of cAMP-dependent protein kinase. The activation of FFAR3 could be involved in the production of leptin by adipose tissue, regulation of intestinal immunity and secretion of the PYY peptide and GLP-1 hormone by enteroendocrine cells (Ichimura *et al.*, 2014 ▸).

## Synthesis and crystallization   

**Figure 3:**
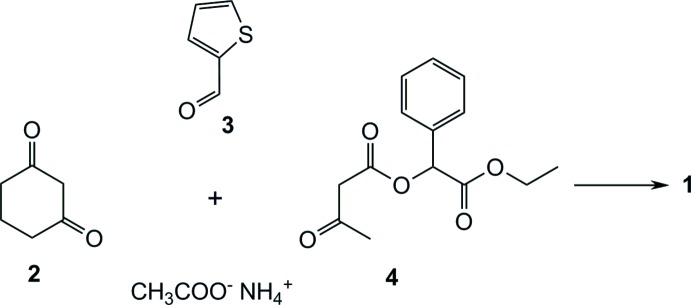
Reaction scheme for the title compound **1**.

4-(Thien-2-yl)-2-methyl-5-oxo-1,4,5,6,7,8-hexa­hydro­quino­line-3-carb­oxy­lic acid eth­oxy­carbonyl­phenyl­methyl­ester **1** was synthesized according to the scheme in Fig. 3 ▸ as follows. A solution of 1.12 g (10 mmol) of thio­phene-2-carbaldehyde **3**, 2.64 g (10 mmol) of 3-oxobutyric acid **4** and 2.3 g (30 mmol) of ammonium acetate in 15 mL of ethanol was stirred at room temperature. After 10 min, 1.12 g (10 mmol) of cyclo­hexane-1,3-dione **2** and 10 drops of acetic acid were added. The reaction mixture was stirred overnight and the resulting precipitate was filtered off and washed with 50% ethanol. After crystallization from ethanol, 2.5 g (55.4%) of compound **1** was obtained. m.p. 494–496 K. ^1^H NMR (400 MHz, CDCl_3_) δ, ppm: 1.13 (3H, *t*, *J* = 7.0 Hz, CH_3_), 1.88–2.00 (2H, *m*, CH_2_), 2.24–2.36 (2H, *m*, CH_2_), 2.37 (3H, *s*, CH_3_), 2.38–2.48 (2H, *m*, CH_2_), 3.95–4.23 (2H, *m*, CH_2_), 5.47 (1H, *s*, CH), 5.88 (1H, *s*, CH), 6.39 (1H, *s*, NH), 6.86 (1H, *dd*, *J* = 5.1, 3.5 Hz, H^Th^), 6.96 (1H, *dt*, *J* = 3.6, 1.0 Hz, H^Th^), 7.04 (1H, *dd*, *J* = 5.1, 1.3 Hz, H^Th^), 7.30–7.35 (3H, *m*, 3 H^Ar^), 7.36–7.42 (2H, *m*, 2 H^Ar^). LC–MS (ESI), *m*/*z*: 450 ([*M* − H]^−^, 100%). Analysis calculated for C_25_H_25_NO_5_S: C, 66.50; H, 5.58; N, 3.10. Found C, 66.23; H, 5.70; N, 3.00.

## Refinement   

Crystal data, data collection and structure refinement details are summarized in Table 2 ▸. All hydrogen atoms bonded to carbon atoms were placed in calculated positions and included as riding contributions in the final stages of refinement [C*sp* ^3^—H = 0.95–1.00 Å with *U*_iso_(H) = 1.2*U*_eq_(C) for methine and methyl­ene groups, and *U*_iso_(H) = 1.5*U*_eq_(C) for methyl groups]. The hydrogen atom bonded to the nitro­gen atom was identified as the strongest peak in the electron-density difference map and was refined isotropically. There is a two-component disorder in the thienyl group with the ring assuming two positions with opposite orientations. The two orientations were refined as rigid groups using an accurate determination of the geometry of the thienyl group taken from CSD structure UWIYUW (Anil *et al.*, 2016 ▸) as the model. Refinement of the group occupation factor (the second free variable in the FVAR instruction of *SHELXL*) gave the value of 0.7220 (19).

## Supplementary Material

Crystal structure: contains datablock(s) I, global. DOI: 10.1107/S2056989018014251/mw2138sup1.cif

Supporting information file. DOI: 10.1107/S2056989018014251/mw2138Isup3.cdx

Supporting information file. DOI: 10.1107/S2056989018014251/mw2138Isup4.cdx

CCDC reference: 1872194

Additional supporting information:  crystallographic information; 3D view; checkCIF report

## Figures and Tables

**Table 1:** Hydrogen-bond geometry (Å, °)

*D*—H⋯*A*	*D*—H	H⋯*A*	*D*⋯*A*	*D*—H⋯*A*
N1—H1⋯O5^i^	0.89 (3)	1.91 (3)	2.794 (3)	171 (3)

Symmetry code: (i) 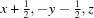.

**Table 2:** Experimental details

Crystal data	
Chemical formula	C_25_H_25_NO_5_S
*M*_r_	451.52
Crystal system, space group	Monoclinic, *P*2_1_/*a*
Temperature (K)	190
*a*, *b*, *c* (Å)	13.9560 (4), 8.2829 (2), 19.9532 (6)
β (°)	95.475 (1)
*V* (Å^3^)	2295.99 (11)
*Z*	4
Radiation type	Mo *K*α
μ (mm^−1^)	0.18
Crystal size (mm)	0.3 × 0.2 × 0.05

Data collection	
Diffractometer	Nonius KappaCCD
No. of measured, independent and observed [*I* > 2σ(*I*)] reflections	16429, 5205, 2784
*R*_int_	0.083
(sin θ/λ)_max_ (Å^−1^)	0.649

Refinement	
*R*[*F* ^2^ > 2σ(*F* ^2^)], *wR*(*F* ^2^), *S*	0.065, 0.166, 1.03
No. of reflections	5205
No. of parameters	290
No. of restraints	27
H-atom treatment	H atoms treated by a mixture of independent and constrained refinement
Δρ_max_, Δρ_min_ (e Å^−3^)	0.70, −0.64

Computer programs: *COLLECT* (Bruker, 2001 ▸), *HKL* *SCALEPACK* and *DENZO* (Otwinowski & Minor, 1997 ▸), *SIR2004* (Burla *et al.*, 2005 ▸), *SHELXL2018/1* (Sheldrick, 2015 ▸), *ORTEP-3 for Windows* (Farrugia, 2012 ▸) and *publCIF* (Westrip, 2010 ▸).

## supplementary crystallographic information

### Crystal data

**Table:**

C_25_H_25_NO_5_S	*F*(000) = 952
*M**_r_* = 451.52	*D*_x_ = 1.306 Mg m^−^^3^
Monoclinic, *P*2_1_/*a*	Mo *K*α radiation, λ = 0.71073 Å
*a* = 13.9560 (4) Å	Cell parameters from 25734 reflections
*b* = 8.2829 (2) Å	θ = 1.0–27.5°
*c* = 19.9532 (6) Å	µ = 0.18 mm^−^^1^
β = 95.475 (1)°	*T* = 190 K
*V* = 2295.99 (11) Å^3^	Plate, colourless
*Z* = 4	0.3 × 0.2 × 0.05 mm

### Data collection

**Table:**

Nonius KappaCCD diffractometer	*R*_int_ = 0.083
CCD scans	θ_max_ = 27.5°, θ_min_ = 2.1°
16429 measured reflections	*h* = −18→17
5205 independent reflections	*k* = −10→9
2784 reflections with *I* > 2σ(*I*)	*l* = −25→25

### Refinement

**Table:**

Refinement on *F*^2^	Primary atom site location: structure-invariant direct methods
Least-squares matrix: full	Secondary atom site location: difference Fourier map
*R*[*F*^2^ > 2σ(*F*^2^)] = 0.065	Hydrogen site location: mixed
*wR*(*F*^2^) = 0.166	H atoms treated by a mixture of independent and constrained refinement
*S* = 1.03	*w* = 1/[σ^2^(*F*_o_^2^) + (0.0734*P*)^2^ + 0.4915*P*] where *P* = (*F*_o_^2^ + 2*F*_c_^2^)/3
5205 reflections	(Δ/σ)_max_ < 0.001
290 parameters	Δρ_max_ = 0.70 e Å^−^^3^
27 restraints	Δρ_min_ = −0.64 e Å^−^^3^

### Special details

**Table:**

Geometry. All esds (except the esd in the dihedral angle between two l.s. planes) are estimated using the full covariance matrix. The cell esds are taken into account individually in the estimation of esds in distances, angles and torsion angles; correlations between esds in cell parameters are only used when they are defined by crystal symmetry. An approximate (isotropic) treatment of cell esds is used for estimating esds involving l.s. planes.

### Fractional atomic coordinates and isotropic or equivalent isotropic displacement parameters (Å^2^)

**Table:**

	*x*	*y*	*z*	*U*_iso_*/*U*_eq_	Occ. (<1)
O1	0.75161 (15)	0.4350 (2)	0.57527 (10)	0.0476 (5)	
O2	0.85597 (15)	0.2267 (2)	0.58740 (10)	0.0491 (6)	
O3	0.90570 (12)	0.3165 (2)	0.71788 (9)	0.0325 (4)	
O4	0.75961 (13)	0.2072 (2)	0.72353 (9)	0.0381 (5)	
O5	0.71302 (13)	−0.3042 (2)	0.83280 (10)	0.0406 (5)	
N1	1.02122 (16)	−0.0942 (3)	0.82113 (12)	0.0339 (6)	
H1	1.084 (2)	−0.117 (3)	0.8230 (14)	0.042 (8)*	
C1	0.6277 (3)	0.2471 (5)	0.53168 (18)	0.0680 (10)	
H1A	0.579524	0.304182	0.553506	0.102*	
H1B	0.656522	0.165993	0.561506	0.102*	
H1C	0.598542	0.196772	0.491455	0.102*	
C2	0.7039 (3)	0.3638 (4)	0.51375 (15)	0.0554 (9)	
H2A	0.750855	0.307788	0.489403	0.067*	
H2B	0.674782	0.448237	0.484926	0.067*	
C3	0.8232 (2)	0.3498 (3)	0.60713 (14)	0.0374 (7)	
C4	0.8592 (2)	0.4337 (3)	0.67260 (13)	0.0359 (7)	
H4	0.804706	0.481625	0.692952	0.043*	
C5	0.84575 (19)	0.2011 (3)	0.73912 (13)	0.0285 (6)	
C6	0.89291 (17)	0.0766 (3)	0.78203 (12)	0.0256 (6)	
C7	0.8235 (2)	−0.0249 (3)	0.81920 (18)	0.0248 (6)	0.7220 (19)
H7	0.764037	−0.038227	0.789419	0.030*	0.7220 (19)
C7A	0.8263 (4)	−0.0218 (4)	0.8227 (3)	0.0248 (6)	0.2780 (19)
H7A	0.762752	−0.028573	0.797242	0.030*	0.2780 (19)
C8	0.86523 (17)	−0.1902 (3)	0.83423 (12)	0.0251 (6)	
C9	0.96138 (18)	−0.2152 (3)	0.83726 (13)	0.0295 (6)	
C10	0.98829 (17)	0.0443 (3)	0.78774 (13)	0.0287 (6)	
C11	1.06707 (18)	0.1369 (3)	0.75901 (16)	0.0390 (7)	
H11A	1.062770	0.248735	0.770943	0.059*	
H11B	1.060541	0.126373	0.710874	0.059*	
H11C	1.128378	0.094786	0.776803	0.059*	
C12	1.00698 (19)	−0.3764 (3)	0.85434 (15)	0.0379 (7)	
H12A	1.070133	−0.360302	0.878193	0.046*	
H12B	1.015047	−0.435159	0.813211	0.046*	
C13	0.9449 (2)	−0.4740 (3)	0.89777 (16)	0.0414 (7)	
H13A	0.971046	−0.582158	0.903550	0.050*	
H13B	0.946104	−0.424442	0.941887	0.050*	
C14	0.84209 (19)	−0.4835 (3)	0.86628 (15)	0.0372 (7)	
H14A	0.839455	−0.555513	0.827814	0.045*	
H14B	0.802365	−0.529681	0.898707	0.045*	
C15	0.80077 (18)	−0.3218 (3)	0.84359 (13)	0.0286 (6)	
C16	0.79862 (18)	0.0618 (6)	0.88204 (13)	0.0268 (8)	0.7220 (19)
C17	0.71371 (14)	0.1323 (5)	0.89469 (16)	0.0504 (3)	0.7220 (19)
H17	0.659559	0.135793	0.863710	0.060*	0.7220 (19)
C18	0.71712 (14)	0.1995 (4)	0.96007 (17)	0.0507 (11)	0.7220 (19)
H18	0.664822	0.250935	0.976335	0.061*	0.7220 (19)
C19	0.80236 (17)	0.1825 (5)	0.99655 (12)	0.0566 (14)	0.7220 (19)
H19	0.816290	0.219499	1.040409	0.068*	0.7220 (19)
S1	0.88191 (9)	0.0831 (2)	0.95088 (8)	0.0504 (3)	0.7220 (19)
C16A	0.8149 (5)	0.051 (2)	0.8910 (5)	0.0268 (8)	0.2780 (19)
C17A	0.8822 (4)	0.0718 (18)	0.9447 (6)	0.0504 (3)	0.2780 (19)
H17A	0.944587	0.031398	0.946420	0.060*	0.2780 (19)
C18A	0.8467 (5)	0.1615 (13)	0.9974 (4)	0.0507 (11)	0.2780 (19)
H18A	0.884048	0.186414	1.037172	0.061*	0.2780 (19)
C19A	0.7546 (5)	0.2074 (13)	0.9850 (3)	0.0566 (14)	0.2780 (19)
H19A	0.720689	0.267213	1.014343	0.068*	0.2780 (19)
S1A	0.7081 (2)	0.1401 (5)	0.90743 (15)	0.0504 (3)	0.2780 (19)
C20	0.9317 (2)	0.5633 (3)	0.66095 (14)	0.0394 (7)	
C21	0.9020 (2)	0.7238 (3)	0.65493 (14)	0.0431 (8)	
H21	0.838704	0.751127	0.660639	0.052*	
C22	0.9669 (3)	0.8425 (4)	0.64045 (16)	0.0545 (9)	
H22	0.946545	0.949129	0.635671	0.065*	
C23	1.0606 (3)	0.8041 (4)	0.63313 (18)	0.0646 (10)	
H23	1.103624	0.884474	0.623274	0.077*	
C24	1.0917 (3)	0.6457 (4)	0.6404 (2)	0.0700 (11)	
H24	1.155706	0.619703	0.636399	0.084*	
C25	1.0266 (2)	0.5263 (4)	0.65351 (18)	0.0563 (9)	
H25	1.047088	0.419597	0.657395	0.068*	

### Atomic displacement parameters (Å^2^)

**Table:**

	*U*^11^	*U*^22^	*U*^33^	*U*^12^	*U*^13^	*U*^23^
O1	0.0618 (14)	0.0392 (11)	0.0398 (12)	0.0095 (10)	−0.0058 (10)	0.0052 (9)
O2	0.0655 (15)	0.0385 (12)	0.0443 (13)	0.0115 (10)	0.0104 (11)	−0.0041 (10)
O3	0.0325 (10)	0.0299 (10)	0.0360 (11)	0.0012 (8)	0.0080 (8)	0.0095 (8)
O4	0.0284 (11)	0.0410 (11)	0.0451 (12)	0.0073 (8)	0.0045 (9)	0.0111 (9)
O5	0.0237 (11)	0.0410 (11)	0.0574 (13)	−0.0076 (9)	0.0057 (9)	−0.0006 (10)
N1	0.0174 (12)	0.0310 (12)	0.0530 (15)	0.0010 (10)	0.0018 (10)	0.0108 (11)
C1	0.083 (3)	0.074 (2)	0.046 (2)	−0.014 (2)	−0.0019 (19)	0.0048 (18)
C2	0.075 (2)	0.0536 (19)	0.0348 (18)	−0.0023 (18)	−0.0074 (16)	0.0111 (15)
C3	0.0494 (18)	0.0321 (15)	0.0321 (15)	0.0035 (14)	0.0107 (14)	0.0079 (13)
C4	0.0452 (17)	0.0298 (14)	0.0335 (15)	0.0095 (13)	0.0078 (13)	0.0084 (12)
C5	0.0294 (15)	0.0269 (13)	0.0305 (14)	0.0040 (12)	0.0096 (12)	0.0022 (11)
C6	0.0241 (13)	0.0256 (13)	0.0273 (13)	−0.0008 (11)	0.0031 (11)	0.0006 (11)
C7	0.0174 (13)	0.0259 (13)	0.0305 (14)	0.0003 (10)	0.0001 (11)	0.0013 (11)
C7A	0.0174 (13)	0.0259 (13)	0.0305 (14)	0.0003 (10)	0.0001 (11)	0.0013 (11)
C8	0.0220 (13)	0.0260 (13)	0.0278 (14)	−0.0026 (11)	0.0047 (10)	0.0002 (11)
C9	0.0245 (14)	0.0250 (13)	0.0392 (16)	−0.0018 (11)	0.0033 (12)	0.0018 (11)
C10	0.0255 (14)	0.0251 (13)	0.0357 (15)	−0.0024 (11)	0.0038 (11)	0.0030 (11)
C11	0.0255 (15)	0.0347 (15)	0.0577 (19)	−0.0005 (12)	0.0083 (13)	0.0102 (14)
C12	0.0301 (15)	0.0279 (14)	0.0555 (19)	0.0046 (12)	0.0026 (13)	0.0071 (13)
C13	0.0425 (17)	0.0264 (14)	0.0556 (19)	0.0040 (13)	0.0064 (14)	0.0101 (13)
C14	0.0397 (16)	0.0267 (14)	0.0468 (17)	−0.0050 (12)	0.0119 (13)	−0.0012 (12)
C15	0.0283 (15)	0.0301 (14)	0.0276 (14)	−0.0037 (12)	0.0048 (11)	−0.0031 (11)
C16	0.0268 (18)	0.0221 (15)	0.0319 (18)	0.0046 (15)	0.0052 (14)	0.0060 (15)
C17	0.0604 (6)	0.0523 (6)	0.0378 (6)	−0.0114 (4)	0.0017 (4)	−0.0017 (4)
C18	0.069 (3)	0.036 (2)	0.053 (3)	−0.008 (2)	0.033 (2)	−0.008 (2)
C19	0.085 (4)	0.050 (2)	0.038 (2)	−0.016 (3)	0.020 (2)	−0.0116 (18)
S1	0.0604 (6)	0.0523 (6)	0.0378 (6)	−0.0114 (4)	0.0017 (4)	−0.0017 (4)
C16A	0.0268 (18)	0.0221 (15)	0.0319 (18)	0.0046 (15)	0.0052 (14)	0.0060 (15)
C17A	0.0604 (6)	0.0523 (6)	0.0378 (6)	−0.0114 (4)	0.0017 (4)	−0.0017 (4)
C18A	0.069 (3)	0.036 (2)	0.053 (3)	−0.008 (2)	0.033 (2)	−0.008 (2)
C19A	0.085 (4)	0.050 (2)	0.038 (2)	−0.016 (3)	0.020 (2)	−0.0116 (18)
S1A	0.0604 (6)	0.0523 (6)	0.0378 (6)	−0.0114 (4)	0.0017 (4)	−0.0017 (4)
C20	0.058 (2)	0.0305 (15)	0.0318 (15)	−0.0001 (14)	0.0137 (14)	0.0037 (12)
C21	0.066 (2)	0.0301 (15)	0.0326 (16)	0.0020 (15)	−0.0012 (14)	0.0039 (12)
C22	0.087 (3)	0.0302 (16)	0.0449 (19)	−0.0051 (18)	0.0009 (18)	0.0012 (14)
C23	0.092 (3)	0.0387 (19)	0.067 (2)	−0.0210 (19)	0.026 (2)	−0.0016 (17)
C24	0.070 (3)	0.047 (2)	0.099 (3)	−0.0094 (18)	0.041 (2)	0.002 (2)
C25	0.064 (2)	0.0356 (17)	0.075 (2)	−0.0012 (16)	0.0362 (19)	0.0043 (16)

### Geometric parameters (Å, º)

**Table:**

C1—C2	1.505 (5)	C14—C13	1.513 (4)
C1—H1A	0.9600	C14—H14A	0.9700
C1—H1B	0.9600	C14—H14B	0.9700
C1—H1C	0.9600	C13—H13A	0.9700
O1—C3	1.334 (3)	C13—H13B	0.9700
O1—C2	1.463 (4)	C7—C16	1.514 (3)
C5—O4	1.214 (3)	C7—H7	0.9800
C5—O3	1.364 (3)	C16—C17	1.3662
C5—C6	1.456 (3)	C16—S1	1.7216
O5—C15	1.232 (3)	C17—C18	1.4147
C4—O3	1.438 (3)	C17—H17	0.9300
C4—C20	1.508 (4)	C18—C19	1.3415
C4—C3	1.522 (4)	C18—H18	0.9300
C4—H4	0.9800	C19—S1	1.7128
C3—O2	1.200 (3)	C19—H19	0.9300
N1—C9	1.363 (3)	C7A—C16A	1.514 (4)
N1—C10	1.383 (3)	C7A—H7A	0.9800
N1—H1	0.89 (3)	C16A—C17A	1.3662
C2—H2A	0.9700	C16A—S1A	1.7217
C2—H2B	0.9700	C17A—C18A	1.4147
C6—C10	1.352 (3)	C17A—H17A	0.9300
C6—C7A	1.527 (4)	C18A—C19A	1.3415
C6—C7	1.527 (3)	C18A—H18A	0.9300
C9—C8	1.353 (3)	C19A—S1A	1.7129
C9—C12	1.504 (3)	C19A—H19A	0.9300
C8—C15	1.437 (3)	C20—C25	1.380 (4)
C8—C7A	1.507 (4)	C20—C21	1.394 (4)
C8—C7	1.507 (3)	C21—C22	1.386 (4)
C11—C10	1.498 (4)	C21—H21	0.9300
C11—H11A	0.9600	C22—C23	1.367 (5)
C11—H11B	0.9600	C22—H22	0.9300
C11—H11C	0.9600	C23—C24	1.385 (5)
C12—C13	1.516 (4)	C23—H23	0.9300
C12—H12A	0.9700	C24—C25	1.384 (4)
C12—H12B	0.9700	C24—H24	0.9300
C14—C15	1.510 (4)	C25—H25	0.9300
			
C2—C1—H1A	109.5	C14—C13—H13A	109.4
C2—C1—H1B	109.5	C12—C13—H13A	109.4
H1A—C1—H1B	109.5	C14—C13—H13B	109.4
C2—C1—H1C	109.5	C12—C13—H13B	109.4
H1A—C1—H1C	109.5	H13A—C13—H13B	108.0
H1B—C1—H1C	109.5	O5—C15—C8	120.8 (2)
C3—O1—C2	116.5 (2)	O5—C15—C14	120.2 (2)
O4—C5—O3	120.9 (2)	C8—C15—C14	119.1 (2)
O4—C5—C6	124.1 (2)	C8—C7—C16	112.4 (3)
O3—C5—C6	115.0 (2)	C8—C7—C6	110.3 (2)
O3—C4—C20	108.0 (2)	C16—C7—C6	110.3 (3)
O3—C4—C3	108.8 (2)	C8—C7—H7	107.9
C20—C4—C3	111.4 (2)	C16—C7—H7	107.9
O3—C4—H4	109.5	C6—C7—H7	107.9
C20—C4—H4	109.5	C17—C16—C7	128.87 (18)
C3—C4—H4	109.5	C17—C16—S1	109.9
C5—O3—C4	114.5 (2)	C7—C16—S1	121.23 (18)
O2—C3—O1	125.8 (3)	C16—C17—C18	112.6
O2—C3—C4	124.4 (3)	C16—C17—H17	123.7
O1—C3—C4	109.8 (2)	C18—C17—H17	123.7
C9—N1—C10	122.8 (2)	C19—C18—C17	114.2
C9—N1—H1	117.0 (18)	C19—C18—H18	122.9
C10—N1—H1	118.4 (19)	C17—C18—H18	122.9
O1—C2—C1	109.5 (3)	C18—C19—S1	110.4
O1—C2—H2A	109.8	C18—C19—H19	124.8
C1—C2—H2A	109.8	S1—C19—H19	124.8
O1—C2—H2B	109.8	C19—S1—C16	92.9
C1—C2—H2B	109.8	C8—C7A—C16A	107.4 (6)
H2A—C2—H2B	108.2	C8—C7A—C6	110.3 (3)
C10—C6—C5	125.1 (2)	C16A—C7A—C6	112.7 (8)
C10—C6—C7A	119.6 (3)	C8—C7A—H7A	108.8
C5—C6—C7A	115.2 (3)	C16A—C7A—H7A	108.8
C10—C6—C7	121.0 (2)	C6—C7A—H7A	108.8
C5—C6—C7	113.8 (2)	C17A—C16A—C7A	129.3 (4)
C8—C9—N1	120.3 (2)	C17A—C16A—S1A	109.9
C8—C9—C12	122.9 (2)	C7A—C16A—S1A	120.6 (4)
N1—C9—C12	116.8 (2)	C16A—C17A—C18A	112.6
C9—C8—C15	120.6 (2)	C16A—C17A—H17A	123.7
C9—C8—C7A	119.4 (3)	C18A—C17A—H17A	123.7
C15—C8—C7A	120.1 (3)	C19A—C18A—C17A	114.2
C9—C8—C7	120.7 (2)	C19A—C18A—H18A	122.9
C15—C8—C7	118.7 (2)	C17A—C18A—H18A	122.9
C10—C11—H11A	109.5	C18A—C19A—S1A	110.4
C10—C11—H11B	109.5	C18A—C19A—H19A	124.8
H11A—C11—H11B	109.5	S1A—C19A—H19A	124.8
C10—C11—H11C	109.5	C19A—S1A—C16A	92.9
H11A—C11—H11C	109.5	C25—C20—C21	118.9 (3)
H11B—C11—H11C	109.5	C25—C20—C4	121.5 (2)
C6—C10—N1	118.9 (2)	C21—C20—C4	119.6 (3)
C6—C10—C11	128.2 (2)	C22—C21—C20	120.0 (3)
N1—C10—C11	112.7 (2)	C22—C21—H21	120.0
C9—C12—C13	110.5 (2)	C20—C21—H21	120.0
C9—C12—H12A	109.5	C23—C22—C21	120.5 (3)
C13—C12—H12A	109.5	C23—C22—H22	119.7
C9—C12—H12B	109.5	C21—C22—H22	119.7
C13—C12—H12B	109.5	C22—C23—C24	120.1 (3)
H12A—C12—H12B	108.1	C22—C23—H23	119.9
C15—C14—C13	113.5 (2)	C24—C23—H23	119.9
C15—C14—H14A	108.9	C25—C24—C23	119.5 (4)
C13—C14—H14A	108.9	C25—C24—H24	120.3
C15—C14—H14B	108.9	C23—C24—H24	120.3
C13—C14—H14B	108.9	C20—C25—C24	121.0 (3)
H14A—C14—H14B	107.7	C20—C25—H25	119.5
C14—C13—C12	111.2 (2)	C24—C25—H25	119.5
			
O4—C5—O3—C4	−4.6 (3)	C15—C8—C7—C6	−155.5 (2)
C6—C5—O3—C4	176.8 (2)	C10—C6—C7—C8	−24.2 (4)
C20—C4—O3—C5	172.9 (2)	C5—C6—C7—C8	153.0 (2)
C3—C4—O3—C5	−66.1 (3)	C10—C6—C7—C16	100.6 (3)
C2—O1—C3—O2	5.5 (4)	C5—C6—C7—C16	−82.3 (3)
C2—O1—C3—C4	−175.4 (2)	C8—C7—C16—C17	−127.5 (3)
O3—C4—C3—O2	−23.9 (4)	C6—C7—C16—C17	109.0 (4)
C20—C4—C3—O2	95.0 (3)	C8—C7—C16—S1	52.4 (5)
O3—C4—C3—O1	157.0 (2)	C6—C7—C16—S1	−71.1 (4)
C20—C4—C3—O1	−84.1 (3)	C7—C16—C17—C18	178.9 (6)
C3—O1—C2—C1	84.0 (3)	S1—C16—C17—C18	−1.1
O4—C5—C6—C10	163.8 (3)	C16—C17—C18—C19	0.5
O3—C5—C6—C10	−17.6 (4)	C17—C18—C19—S1	0.3
O4—C5—C6—C7A	−16.1 (5)	C18—C19—S1—C16	−0.7
O3—C5—C6—C7A	162.5 (3)	C17—C16—S1—C19	1.0
O4—C5—C6—C7	−13.2 (4)	C7—C16—S1—C19	−178.9 (5)
O3—C5—C6—C7	165.4 (2)	C9—C8—C7A—C16A	−94.3 (6)
C10—N1—C9—C8	−14.3 (4)	C15—C8—C7A—C16A	84.1 (7)
C10—N1—C9—C12	162.4 (3)	C9—C8—C7A—C6	28.9 (6)
N1—C9—C8—C15	172.8 (2)	C15—C8—C7A—C6	−152.7 (3)
C12—C9—C8—C15	−3.7 (4)	C10—C6—C7A—C8	−30.1 (6)
N1—C9—C8—C7A	−8.7 (5)	C5—C6—C7A—C8	149.8 (4)
C12—C9—C8—C7A	174.7 (4)	C10—C6—C7A—C16A	89.9 (5)
N1—C9—C8—C7	−5.6 (4)	C5—C6—C7A—C16A	−90.2 (5)
C12—C9—C8—C7	177.9 (3)	C8—C7A—C16A—C17A	56.8 (10)
C5—C6—C10—N1	−169.0 (2)	C6—C7A—C16A—C17A	−64.8 (9)
C7A—C6—C10—N1	10.9 (5)	C8—C7A—C16A—S1A	−129.4 (9)
C7—C6—C10—N1	7.8 (4)	C6—C7A—C16A—S1A	108.9 (11)
C5—C6—C10—C11	7.7 (4)	C7A—C16A—C17A—C18A	173.3 (17)
C7A—C6—C10—C11	−172.4 (4)	S1A—C16A—C17A—C18A	−1.1
C7—C6—C10—C11	−175.5 (3)	C16A—C17A—C18A—C19A	0.5
C9—N1—C10—C6	13.0 (4)	C17A—C18A—C19A—S1A	0.3
C9—N1—C10—C11	−164.1 (3)	C18A—C19A—S1A—C16A	−0.7
C8—C9—C12—C13	−27.0 (4)	C17A—C16A—S1A—C19A	1.0
N1—C9—C12—C13	156.3 (2)	C7A—C16A—S1A—C19A	−173.9 (15)
C15—C14—C13—C12	−48.6 (3)	O3—C4—C20—C25	39.8 (4)
C9—C12—C13—C14	51.9 (3)	C3—C4—C20—C25	−79.7 (3)
C9—C8—C15—O5	−171.0 (2)	O3—C4—C20—C21	−142.7 (2)
C7A—C8—C15—O5	10.5 (5)	C3—C4—C20—C21	97.8 (3)
C7—C8—C15—O5	7.4 (4)	C25—C20—C21—C22	1.2 (4)
C9—C8—C15—C14	8.4 (4)	C4—C20—C21—C22	−176.4 (3)
C7A—C8—C15—C14	−170.1 (4)	C20—C21—C22—C23	−1.2 (5)
C7—C8—C15—C14	−173.2 (3)	C21—C22—C23—C24	−0.2 (5)
C13—C14—C15—O5	−162.0 (3)	C22—C23—C24—C25	1.5 (6)
C13—C14—C15—C8	18.6 (4)	C21—C20—C25—C24	0.1 (5)
C9—C8—C7—C16	−100.6 (3)	C4—C20—C25—C24	177.6 (3)
C15—C8—C7—C16	81.0 (4)	C23—C24—C25—C20	−1.4 (6)
C9—C8—C7—C6	22.9 (4)		

### Hydrogen-bond geometry (Å, º)

**Table:**

*D*—H···*A*	*D*—H	H···*A*	*D*···*A*	*D*—H···*A*
N1—H1···O5^i^	0.89 (3)	1.91 (3)	2.794 (3)	171 (3)

Symmetry code: (i) *x*+1/2, −*y*−1/2, *z*.
